# Spontaneous healing of acute ACL ruptures: rate, prognostic factors and short-term outcome

**DOI:** 10.1007/s00402-022-04701-0

**Published:** 2022-12-14

**Authors:** F. Blanke, K. Trinnes, N. Oehler, W. C. Prall, C. Lutter, T. Tischer, S. Vogt

**Affiliations:** 1 Department of Knee-, Shoulder- and Hip-Surgery and Orthopedic Sports Medicine, Schön Klinik München-Harlaching, Munich, Germany; 2 Department of Orthopedic Surgery, University Rostock, Rostock, Germany; 3 Department of Orthopedic Sports Medicine and Arthroscopic Surgery, Hessing Stiftung Augsburg, Augsburg, Germany; 4 Department of Orthopedic Surgery, University Hospital of Ludwig Maximilian University (LMU), Munich, Germany

**Keywords:** ACL rupture, Conservative, Spontaneous healing, Non-surgical

## Abstract

**Introduction:**

Anterior cruciate ligament (ACL) reconstruction is considered the first line treatment in ACL rupture. However, some patients return to high intensity sport activities and show a normal knee function without ACL reconstruction. Therefore, aim of this study was to evaluate the rate and prognostic factors of spontaneous healing in patients with ACL rupture and the short-term functional outcome.

**Methods:**

The rate, prognostic factors and short-term functional results of spontaneous healing in patients with ACL rupture were evaluated in 381 patients. Morphology of ACL rupture and extent of posterior tibial slope (PTS) were classified by MR- and x-ray imaging. In patients with normal knee stability in anesthesia examination and healed ACL during the arthroscopy 6 weeks after trauma ACL reconstruction was canceled. IKDC -, Tegner Activity Score, KT 1000 testing and radiological characteristics were collected 12 months postoperatively in these patients.

**Results:**

14.17% of the patients with ACL rupture showed a spontaneous healing after 6 weeks. Femoral ACL-rupture (*p < *0.02) with integrity of ligament stump > 50% (*p < *0.001), without bundle separation (*p < *0.001) and decreased PTS (*p < *0.001) was found significantly more often in patients with a spontaneous healed ACL. The average IKDC score was high at 84,63 in patients with healed ACL at 1 year follow-up, but KT 1000 testing was inferior compared to non-injured side.

**Conclusion:**

Spontaneous healing of a ruptured ACL happened in 14% of the patients. Especially in low-demand patients with femoral single bundle lesions without increased posterior tibial slope delayed ACL surgery should be considered to await the possibility for potential spontaneous ACL healing.

## Introduction

Anterior cruciate ligament (ACL) rupture is one of the most frequent injuries in the knee joint [1]. Due to anatomic and minimal-invasive surgical techniques for ACL reconstruction, restoration of previous knee stability can often be achieved and patients are able to return to several sport activities [1–3]. However, the function of the native ACL is complex, and it is still a demanding task to recreate this role in the knee joint by ACL reconstruction [4–8]. Approximately 37–55% of patients do not return to the same activity level after ACL reconstruction compared to the pre-traumatic situation [9, 10]. This fact aroused interest in several studies, which reported good outcomes following conservative treatment of ACL ruptures [11–15]. These results are commonly explained by muscular compensation or activity modification. However, some patients return to high intensity sport activities and show a normal knee function in the clinical examination [13, 16]. That raises the question of possible spontaneous healing of a ruptured ACL. To the best of our knowledge, there are still only a few small sample studies addressing this topic and relevant data is still pending [17, 18]. Therefore, the purpose of the present study was to evaluate the rate and prognostic factors of spontaneous healing in patients with ACL rupture and the short-term functional outcome. We hypothesized that spontaneous healing happens in a considerable number of patients with ACL rupture depending on the rupture pattern and can lead to good functional results.

## Methods

**Fig. 1:**
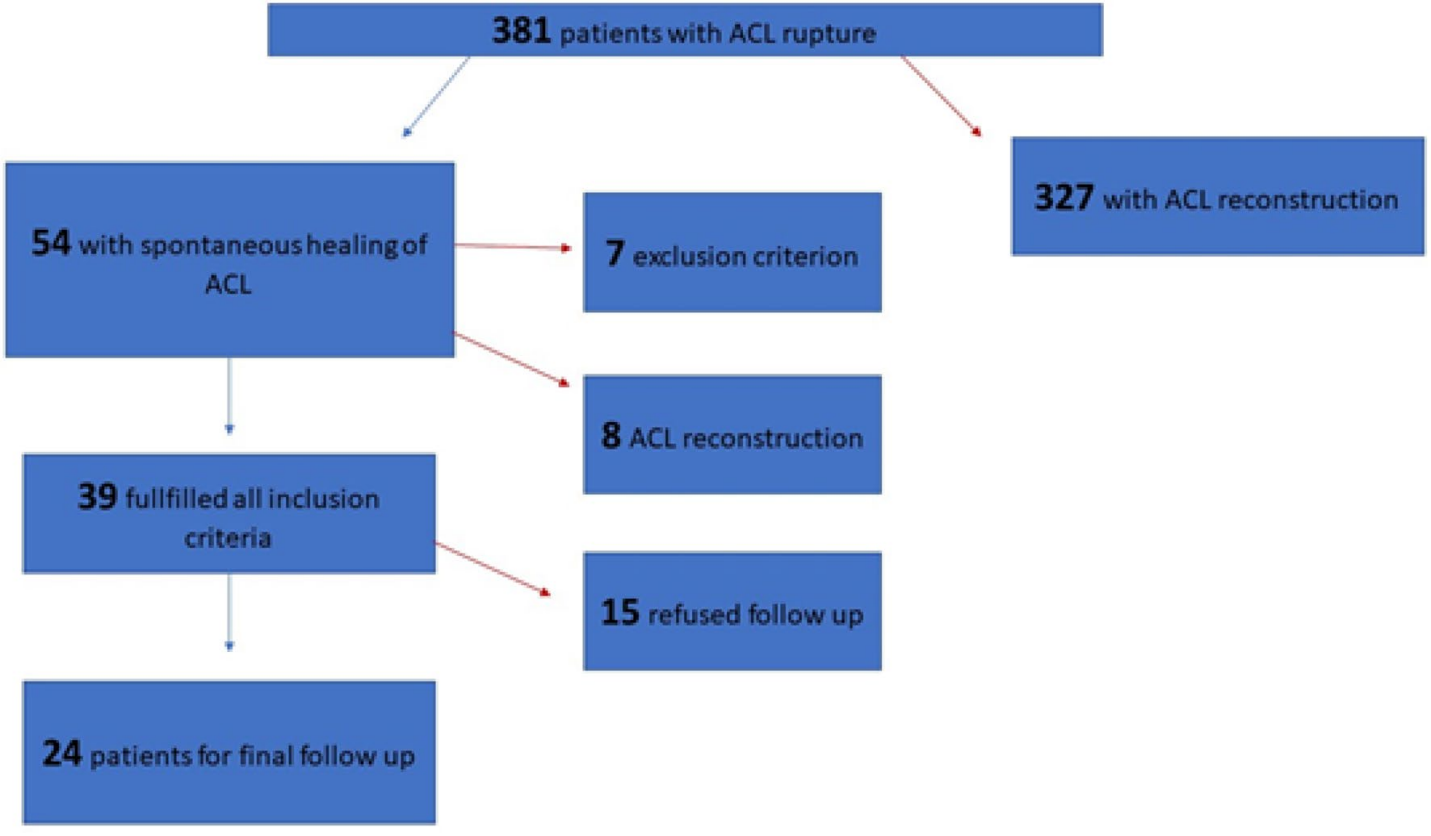
Study enrollment

The study was approved by the institutional review board and conducted according to the Declaration of Helsinki. All patients gave their written and informed consent. From 2018–2019, 438 patients with ACL rupture were treated in our hospital. 381 patients with primary ACL rupture were identified for retrospective evaluation. Diagnosis of ACL rupture was verified by Magnetic Resonance Imaging (MRI) and a positive Lachman test with a side-to-side difference to non-injured knee. All patients initially received an ACL knee support brace with free range of motion for 6 weeks with physical therapy for range of motion exercises and were allowed for pain adapted full weight bearing. After at least 6 weeks (range: 6–9 weeks) a knee arthroscopy was performed in all patients. In patients with negative Lachman- and Pivot-Shift test during anesthesia examination and fully healed ACL in the arthroscopic evaluation, the ACL reconstruction was canceled. These patients were included for follow-up examination at least 12 months postoperatively. Clinical and radiological characteristics were compared between patients with healed ACL (Group A, 33.5 y range: 18–63 y, female: 31.5%) and non-healed ACL (Group B, 27.4 y range: 18–64 y, female: 29.2%). Patients under 18 years, with prior knee injury, complete rupture or clinical instability of the medial collateral ligament (MCL), lateral collateral ligament (LCL) or meniscal root or with any concomitant arthroscopic procedure were excluded. Detailed enrollment is displayed in Fig. 1.

### Surgical technique

All patients were operated at a single institution by three experienced sports orthopedic surgeons. After the knee was examined under general anesthesia with Lachman-test and Pivot-Shift-Test, a routine diagnostic arthroscopic procedure was performed through an anterolateral portal with the tourniquet inflated to 250 mmHg. The ACL was examined with a probe to verify a complete healing of the ACL. A complete healing was defined as a full ligamentous coverage of the footprints and a tight ACL in 20–30° of flexion. If the ACL showed an incomplete healing in arthroscopic evaluation or the Lachman-Test or the Pivot-Shift Test was positive, an ACL reconstruction was performed. A longitudinal incision was made over the pes anserinus to expose the semitendinosus tendon. The tendon was quadrupled and armed with Fiber Wire Sutures (Fa. Arthrex, Naples, Florida). Single-bundle ACL reconstruction was performed by anteromedial drilling for the femoral socket followed by tibial drilling for the tibial socket. The tibial socket preparation was done by transtibial drilling. Femoral fixation was secured in Tight-Rope technique (ACL Tight Rope, Arthrex, Naples, Florida), tibial fixation with a BioComposite Interference screw (Fa. Arthrex, Naples, Florida).

### Radiological examination

#### Classification of ACL rupture

**Fig. 2:**
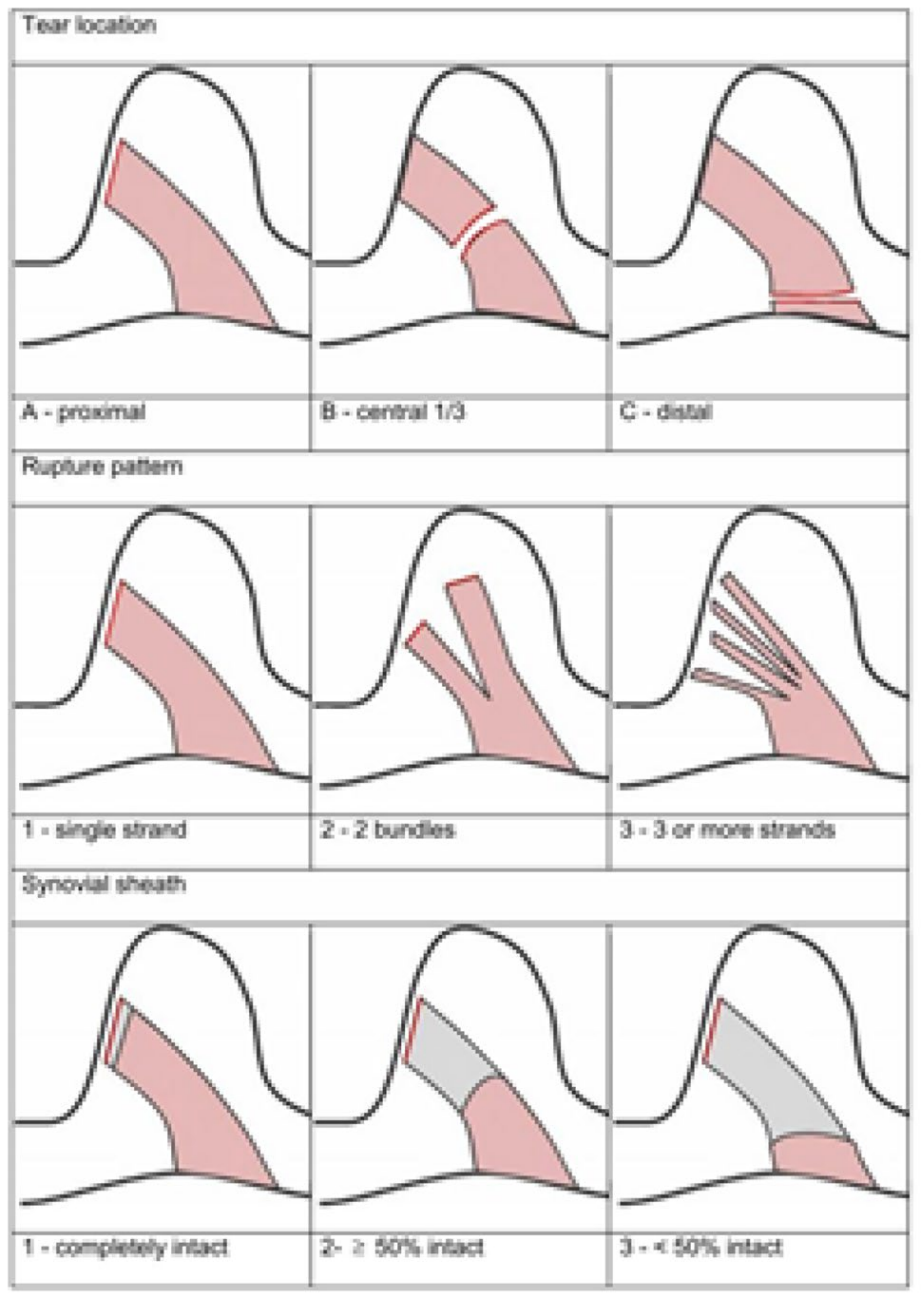
Schematic overview of the classification of ACL ruptures

For ACL rupture classification, three sub-categories were defined according to Henle et al. [19]. The three sub-categories were rupture localization, rupture pattern and integrity of the tibial ligament stump (including the synovial sheath). We classified ruptures as (1) “tibial” if they were between the tibial insertion and about 25% of the total ligament length, (2) “intraligamentary” if they were located between 25 and 75% and (3) “femoral” when found to be > 75% from the tibial insertion. Figure 2 shows a schematic overview of the complete classification. For the definitive classification of the ACL rupture, sagittal and coronal MRI images of the intercondylar region were chosen, in which the ACL was displayed completely. MRI examination was done on a 3.0-Tesla scanner (Avanto; Siemens Medical Systems, Erlangen, Germany). Evaluation of MR images and classification of the ACL rupture was done by two experienced orthopedic surgeons.

#### Measurement of the posterior tibial slope (PTS)

**Fig. 3:**
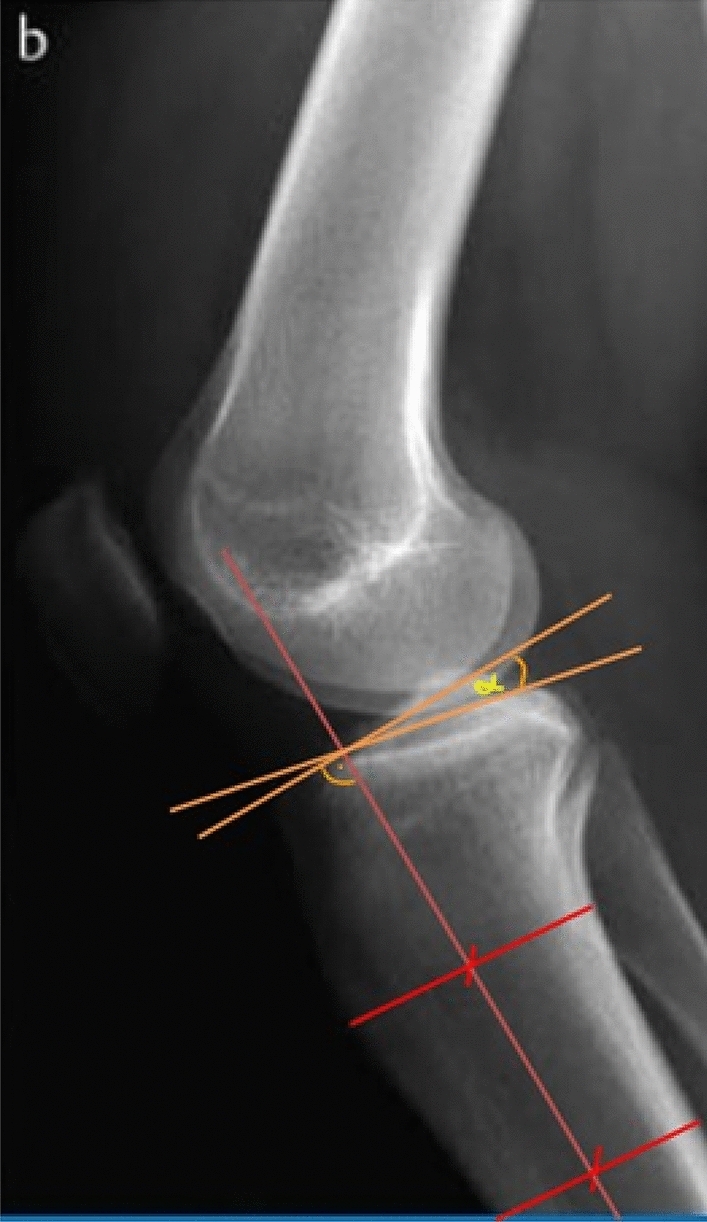
Measurement of the posterior tibial slope (PTS)

The PTS describes the posterior angle between the longitudinal axis of the tibial shaft and the tibial plateau. The PTS was measured on lateral x-rays of the knee joint. The measurement was performed according to the method of Brandon et al. [20] First, the longitudinal axis of the tibia (orange line) was determined. The red lines show the tibial diameter below the tibial tuberosity and at least 5 cm distal. The PTS represents the angle between a perpendicular line to the tibial shaft (blue line) and a tangent to the tibia plateau (green line) (Fig. 3).

### Clinical examination

The follow-up examination of the patients with a spontaneous healed ACL was performed at least 12 months after definite treatment. In all patients, the overall knee function was assessed by the International Knee Documentation Committee (IKDC)—Score and the subjective activity level by the means by the Tegner Activity Scale [21, 22].

The anterior tibial translation was evaluated by a Knee Ligament Arthrometer (KT-1000™, MEDmetric^®^, San Diego, California, U.S.A.)

### Statistical analysis

Continuous variables are presented as mean, standard deviation (SD), maximums and minimums. Categorical variables are presented as percentages. The analysis of categorical variables was done by the Fisher test. For continuous variables the *t* test and the Kruskal–Wallis test was performed. Statistical significance was set at a *p* value of < 0.05. Data was analyzed using statistics program "R" (RStudio, Version 1.2.1335).

## Results

**Fig. 4:**
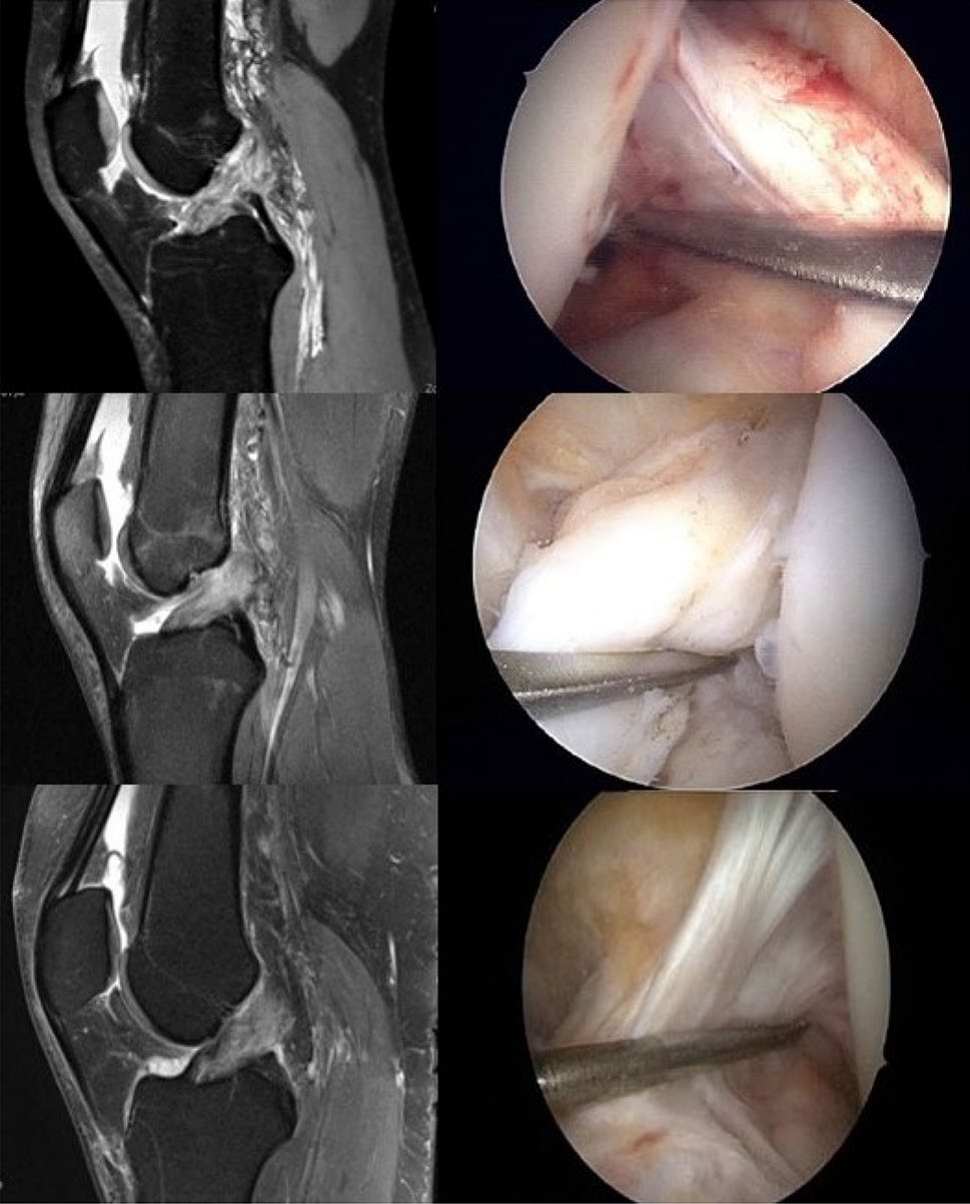
MR imaging after ACL injury and arthroscopic view of healed ACL 6 weeks after injury

54 of 381 patients (14.17%, Group A) with ACL rupture showed stable knees during exam and arthroscopically healed ACL after at least 6 weeks (Fig. 4). However, 7 of these patients were not included due to concomitant knee injuries. 327 of 381 patients (85.83%, Group B) received an ACL reconstruction, because an ACL insufficiency was diagnosed during exam and in the arthroscopy 6 weeks after the ACL injury.

### Classification of ACL rupture

129 of 381 patients showed a femoral ACL rupture (33.85%), 221 patients were classified as intraligamentary (58.01%) und 19 patients showed tibial ACL ruptures (4.99%). In 12 cases a definitive classification was not possible (3.15%).

103 of the 327 patients with ACL reconstruction showed a femoral ACL rupture (31.5%), whereas 26 of the 54 patients with spontaneous healed ACL had a femoral ACL rupture (48.1%).

262 of 381 patients showed a one bundle ACL rupture (68.8%), 60 patients a two bundle (15.7%) und 41 patients showed three or more bundle ACL rupture (10.8%). In 18 cases a definitive classification was not possible (4.7%).

211 of the 327 patients with ACL reconstruction showed a one bundle ACL rupture (64.5%). Whereas 51 of the 54 patients with spontaneous healed ACL had this kind of ACL rupture (94.4%). Therefore, almost all patient with spontaneous ACL healing had a partial ACL rupture.

77 of 381 patients showed a completely intact anterior cruciate ligament and synovial sheath (20.2%), in 187 patients the ligament was intact > 50% (49.1%) and in 99 patients it was intact < 50% (26%). In 18 cases a definitive classification was not possible (4.7%).

52 of the 327 patients with ACL reconstruction showed a completely intact ligament stump (15.9%). Whereas 25 of the 54 patients with spontaneous healed ACL showed this characteristic in the MRI (46%).

Therefore, femoral ACL-rupture (*p < *0.02) with integrity of ligament stump > 50% (*p < *0.001) and without bundle separation (*p < *0.001) was significantly more often in patients with a spontaneous healed ACL (Table 1).

**Table 1:** Patient characteristics

	ACL-reconstruction *n* = 327 [SD]	Spontaneous healing *n* = 54 [SD]	*p* value
Rupture localization			
Femoral	103	26	0.02*
Intraligamentary	195	26	0.14
Tibial	17	2	1
Classification not possible	12	–	
Rupture pattern			
One bundle	211	51	*< *0.001*
Two bundles	57	3	
Three or more bundles	41	–	
Classification not possible	18	–	
Integrity ligament stump			
Completely intact	52	25	*< *0.001*
> 50% intact	161	26	1
< 50% intact	96	3	*< *0.001*
Classification not possible	18	–	
PTS	9.84° [3, 54]	8.16° [2.44]	*< *0.001*
IKDC Score (*n* = 24, FU)	–	84.63 [11.76]	
KT-1000 (mm) (*n* = 24, FU)		Injured side: 9.46 [2.09] Non-injured side: 7.67 [2.33]	*< *0.001*
TAS (*n* = 24, FU)		Before injury: 6.92 [1.02] After injury: 5.79 [1.53]	*< *0.001**

*significant difference (*p* < 0.05)

### Posterior tibial slope (PTS)

PTS was significantly increased in patients with ACL reconstruction (9.84 ± 23.54) compared to the patients with healed ACL (8.16 ± 2.44) (*p < *0.001) (Table 1).

### Follow-up examination

47 patients met the inclusion criteria for the follow-up examination. However, in 8 patients (17%) an ACL reconstruction was necessary due to another knee trauma. Therefore 39 patients were qualified for the examination. 24 of these 39 patients were at last available for the follow-up examinations (12–36 months), the other 15 patients (38.5%) were not interested because of different reasons. The average follow-up examination was 30.4 months postoperative (range: 12–36 months).

The average TAS before injury was 6.92 (± 21.02). In the follow-up examination at least 12 months after arthroscopy, the TAS was significantly decreased to 5.79 (± 21.53) (*p < *0.001).

Therefore, the average difference between the TAS before and after treatment (minimal detectable change, MDC) was 1.13.

KT 1000 testing showed slightly more ap laxity (9.46 ± 22.09 mm vs. 7.67 ± 22.33 mm) in the injured knee compared to the non-injured side e (*p < *0.001). However, 18 of the 24 patients were grouped as normal (0–2 mm) and the 6 other patients were classified as almost normal (3-5 mm).

The IKDC score was high at 84.63 (± 211.76) 12 months postoperative. 16 of the 24 patients (66.7%) reached a score of at least 80 (Table 1).

## Discussion

The main finding of present study was that spontaneous healing of a ruptured ACL happened in 14% of the patients. Especially patients with partial ACL ruptures as femoral one bundle lesions without bony risk factors showed an arthroscopically healed ACL at least 6 weeks after trauma. The overall knee function was satisfactory at midterm follow-up after confirmed ACL healing. However, patients with spontaneously healed ACL showed a significant decreased stability compared to the non-injured side and a reduced Tegner Score compared to the status before the injury.

A stable knee joint with an intact ACL is crucial for a normal knee function and unscathed other knee structures [6, 23, 24]. There is evidence that an ACL insufficiency leads to meniscal damage and cartilage lesions [23, 25]. However, earlier studies showed that conservative and operative treatment of ACL ruptures lead to the same outcome results in the long term follow-up [13, 26]. These studies opened an exhausting discussion about rationality of ACL reconstruction. In the light of current evidence, these results need to be critically considered. In earlier studies almost all patients received a non-anatomic ACL reconstruction with the common biomechanical consequences [11, 13, 25, 26]. Non-anatomic ACL reconstruction leads to increased shear forces within the knee and is inferior compared to anatomic ACL reconstruction [25, 27]. Further, patients with conservative treatment of an ACL rupture regularly make an activity modification and reduce their participation in high demanding sports with only one third of patients returning to pivoting sports [2, 16, 26]. Consequently, recent studies confirmed these theories and showed that an anatomic ACL reconstruction protects the meniscus and can inhibit the development of an osteoarthritis after ACL rupture compared to older techniques [25, 27, 28]. However, the function of the native ACL is complex, and it is still difficult to restore the complete function by ACL reconstruction. Beside the known fact about the two-bundle configuration, the native ACL contains a high nerve supply which enables a distinct proprioception and controls a complex muscle activation while joint motion to protect itself and other knee structures [24, 25, 29–34]. Therefore, the native ACL is superior to any kind of ACL reconstruction and maintaining this ligament must be of highest priority, reflecting the current trend in ACL repair techniques [35–37]

This fact needs to be considered in the treatment of ACL ruptures. Spontaneous healing of a PCL rupture is well known and leads to excellent outcome results [38, 39]. In contrast data about spontaneous healing of an ACL rupture is lacking. Only some sample studies reported the possibility of healing of a ruptured ACL [17, 18, 40].

This study shows that spontaneous healing of ruptured ACL happens in considerable number of patients (approximately 14%). This number is probably even underestimated because patients without subjective instability symptoms and negative Lachman- and Pivot-Shift test in the preoperative examination after six weeks were not indicated for knee arthroscopy and examination under anesthesia. Unfortunately, it was not possible to detect the number of these patients due to the data system of the involved hospital. However, the present study further shows that especially femoral single bundle lesions with an almost intact synovial sheath have a potential for a spontaneous healing. To explain this observation it is useful to remember an earlier study by Hefti et al. [41]. The authors reported in an animal model study that complete ACL lesions did not heal spontaneously, most likely due to the ruptured synovial sheath and the contact of the ligament to the synovial fluid. In contrast, partial ACL lesions showed a complete remodeling after six weeks. Therefore, the femoral one bundle lesions with almost intact synovial sheath of present study most likely represent a partial lesion of the ACL and thus these lesions seem to have a high capability for a spontaneous healing [42, 43]. However, in most of the patients with spontaneously “healed” ACL a complete healing could not be achieved because a slight anterior laxity was noted compared to non-injured side. This could most likely be explained by the fact that the ACL lesions cannot be reliably diagnosed by MRI [44–46]. Some patients with a partial ACL lesion diagnosed by MRI might really have a non-displaced complete lesion and only a scarring rather than a healing is possible. Therefore, in these patients it should rather called a spontaneously scarred ACL. However, in several patients with diagnosed femoral one bundle lesions without bony risk factors a delayed ACL surgery seems still rational to perceive the possibility for a spontaneous ACL healing. Additionally, this treatment concept would also contains the advantage to reduce other complications as arthrofibrosis, which is less likely with delayed ACL surgery [47, 48]. A consideration of the PTS in this context is rational because it was reported as a risk factor for ACL ruptures or re-ruptures in several studies with evidence of high forces on the ligament in individuals with increased PTS values [20, 49–52].

The knee function after a spontaneous ACL healing showed satisfactory results in the present study. The average TAS and the IKDC scores were comparable to the results after ACL reconstruction in several other studies [26–28, 53]. Moreover, the mean KT 1000 side-to-side difference of the patients with spontaneously healed ACL (1.79 mm) was only slightly increased compared to patients after ACL reconstruction in the 12 months follow-up examination (1.2 mm in males and 1.7 mm in females) [54]. However, the nerve supply of the ACL is preserved after a spontaneous healing of the ACL and an ACL reconstruction could still act as a salvage procedure if a re-rupture occurs, which could be a favorable treatment concept in several patients. However, in elite athletes this concept might be limited. The TAS was reduced in patients with spontaneous healed ACL which might be due to activity modification. Moreover, as previously mentioned the ACL healed slightly elongated compared to the uninjured side in most of the patients, which could imply too much instability for high demand sport activities. At least the time-benefit ratio might be too low for the professional sports industry considering the rate of a spontaneous ACL healing and the time interval to get clarity whether a surgery is needed.

This study has several limitations. First, the follow-up sample size was small due to a high number of loss to follow-up and no follow-up was performed in the ACL reconstruction group. Second, there was no follow-up MRI evaluation for verification of sufficiently healed ACL. However, the healing of the ACL was verified by arthroscopy which is very accurate but indeed subjective [55, 56]. Thirdly, a control group with patients who received an early augmented ACL repair would have been interesting to prove superiority of one these treatment options.

## Conclusion

Spontaneous healing of a ruptured ACL happened in 14% of the patients. Especially in low-demand patients with femoral single bundle lesions without increased posterior tibial slope delayed ACL surgery should be considered to await the possibility for potential spontaneous ACL healing.

## Data Availability

Individual patient data will not be available. Individual researchers may contact the corresponding author for access to the original, aggregated and anonymized datasets for research purposes.
